# Correction: Cancer Screening in Appalachia: A Common-Sense Approach

**DOI:** 10.1007/s12529-025-10428-0

**Published:** 2025-12-16

**Authors:** Kimberly M. Kelly, Sabina O. Nduaguba, Randi Shedlosky-Shoemaker, Electra D. Paskett, Nancy Schoenberg, Nicole Yantes

**Affiliations:** 1 University of Tennessee Health Science Center, Memphis, United States; 2 West Virginia University, Morgantown, United States; 3 York College of Pennsylvania, York, United States; 4 The Ohio State University, Columbus, United States; 5 University of Kentucky, Lexington, United States; 6 Genesis HealthCare System, Zanesville, United States

**Correction: International Journal of Behavioral Medicine**

10.1007/s12529-025-10423-5

The original online version of this article was revised: The author Randi Shedlosky-Shoemaker is listed on this article incorrectly as Randi Shedlosky. The author's name has been updated.

